# Comparative Analysis of Subgingival Microbial Profiles in Patients with Periodontal Disease with and Without Depressive Comorbidity

**DOI:** 10.3390/jcm15124402

**Published:** 2026-06-06

**Authors:** Bogdan-Constantin Vasiliu, Diana Tatarciuc, Maria Alexandra Mârțu, Mihaela Monica Scutariu, Doriana Agop Forna, Ionuț Luchian, Sorina Mihaela Solomon

**Affiliations:** Grigore T. Popa University of Medicine and Pharmacy, 16 Universitatii Str., 700115 Iasi, Romania; bogdan.vasiliu@umfiasi.ro (B.-C.V.); maria-alexandra.martu@umfiasi.ro (M.A.M.); mihaela.scutariu@umfiasi.ro (M.M.S.); doriana.agop-forna@umfiasi.ro (D.A.F.); ionut.luchian@umfiasi.ro (I.L.); sorina.solomon@umfiasi.ro (S.M.S.)

**Keywords:** periodontitis, full-mouth disinfection, depression, subgingival microbiota, *Porphyromonas gingivalis*, *Treponema denticola*, real-time PCR

## Abstract

**Background**: Full-mouth disinfection (FMD) has been proposed as an effective non-surgical approach for the management of periodontitis; however, its clinical and microbiological outcomes in patients with depressive comorbidity remain insufficiently explored. **Methods**: This prospective study included 80 patients diagnosed with stage II periodontitis, allocated into two groups based on the presence or absence of depressive disorder. All participants underwent standardized FMD. Clinical parameters, including bleeding on probing and plaque index, together with subgingival bacterial load (total bacterial load, *Aggregatibacter actinomycetemcomitans*, *Porphyromonas gingivalis*, *Treponema denticola*, and *Tannerella forsythia*), were assessed at baseline and 12 weeks post-treatment using quantitative polymerase chain reaction. Microbiological data were log10-transformed prior to statistical analysis. **Results**: Significant reductions in both clinical and microbiological parameters were observed following treatment in both groups. Improvements in bleeding on probing and plaque index were accompanied by a marked decrease in total bacterial load and in the targeted periodontal pathogens. Patients with depressive disorder exhibited a higher baseline microbial burden; however, post-treatment reductions were comparable between groups. Moderate positive correlations were identified between total bacterial load and clinical parameters. **Conclusions**: FMD was associated with substantial short-term improvements in both clinical and microbiological outcomes in patients with stage II periodontitis. Depressive comorbidity did not appear to adversely influence treatment response. These findings support the role of biofilm control as a central component of periodontal therapy across different patient profiles.

## 1. Introduction

Periodontal disease is a chronic, complex, and multifactorial inflammatory condition characterized by progressive destruction of the structures supporting the teeth, such as the gingival tissue, periodontal ligaments, and alveolar bone. Clinically, this pathology manifests itself through loss of attachment, formation of periodontal pockets, and gingival bleeding [1]. Severe forms affect approximately 10–12% of the global population, while mild forms are much more widespread [2,3]. The disruption of the balance between the host’s immune system and the bacterial biofilm is the main triggering factor, amplified by behavioral and systemic factors [4].

Pathogens involved in disease progression include *Aggregatibacter actinomycetemcomitans*, a Gram-negative oral pathobiont associated with periodontal disease and known for virulence mechanisms including leukotoxin production [5], *Porphyromonas gingivalis*, considered a key agent in oral dysbiosis by disrupting the immune response [6], *Treponema denticola*, a spirochete with proteolytic activity and increased motility that promotes tissue invasion [7], and *Tannerella forsythia*, usually detected in deep pockets, responsible for inducing cell apoptosis through specific enzymes [2]. These bacteria colonize multiple niches of the oral cavity, such as the tongue, saliva, tonsils, and mucosa [8,9]. The selected microorganisms were chosen based on their well-established role in periodontitis pathogenesis, particularly their involvement in dysbiosis, tissue destruction, and their frequent detection in moderate periodontal disease [4].

Periodontal treatment includes surgical and non-surgical options, of which non-surgical options aim to mechanically remove biofilm through subgingival instrumentation. The classic procedure, performed on quadrants (scaling and root planing, SRP), can be replaced by a more intensive approach called “full-mouth scaling” (FMS), performed in one or two sessions. By adding antiseptics such as chlorhexidine, the protocol becomes “full-mouth disinfection” (FMD), with the aim of significantly reducing the microbial load in a short period of time [10,11]. Modern treatment options integrate adjuvant therapies such as laser or phototherapy, with proven effects on reducing pathogen levels and improving periodontal parameters, even in severe cases [12,13].

In addition to conventional non-surgical approaches, contemporary periodontal therapy increasingly incorporates individualized and biologically targeted strategies [9]. These include the use of probiotics to modulate the oral microbiome, host modulation therapy with anti-inflammatory agents, and approaches aimed at blocking bacterial virulence factors [1,6]. Furthermore, experimental strategies such as periodontal vaccines and microbiome-directed therapies are being explored as potential tools for long-term disease control. These advances reflect a shift from purely mechanical biofilm removal toward precision-based periodontal care [14].

Depression is a common mental disorder characterized by persistent low mood, anhedonia, and cognitive or somatic symptoms that affect quality of life. Its relationship with periodontal disease is supported by complex physiological mechanisms. Thus, increased glucocorticoid levels reduce the secretion of proinflammatory cytokines, and catecholamines (epinephrine and norepinephrine) stimulate the production of proteolytic enzymes and prostaglandins, which have a destructive effect on periodontal tissues [15,16]. The association of these changes with poor oral hygiene and decreased immunity in depressive states increases susceptibility to carious and periodontal diseases [17]. However, a direct and clear link between psychological and biological factors has not yet been established in the literature.

Antidepressant medication, represented by serotonin reuptake inhibitors (SSRIs), serotonin–norepinephrine reuptake inhibitors (SNRIs), tricyclic antidepressants (TCAs), or monoamine oxidase inhibitors (MAOIs), may indirectly contribute to the development of periodontal disease by reducing salivary flow and altering the oral microbiota, favoring the emergence of microbial imbalances [18].

Depressive disorders may influence the oral microbiome through behavioral (poor hygiene, diet, reduced care), neuroendocrine (HPA axis dysregulation, cortisol and catecholamines), and pharmacological mechanisms. Antidepressants, including SSRIs, SNRIs, TCAs, and MAOIs, are commonly associated with reduced salivary flow, impairing antimicrobial defense and promoting biofilm accumulation and dysbiosis [15,16]. These changes may favor the proliferation of periodontopathogenic species [13]. The selected pathogens, *Aggregatibacter actinomycetemcomitans, Porphyromonas gingivalis*, *Treponema denticola*, and *Tannerella forsythia*, are central to periodontitis: *P. gingivalis* acts as a keystone pathogen driving dysbiosis, *T. denticola* and *T. forsythia* belong to the red complex associated with disease severity, and *A. actinomycetemcomitans* remains relevant through leukotoxin production and host–microbe interactions [14].

In this context, the present study aimed to evaluate the total bacterial load and the amounts of *A. actinomycetemcomitans*, *P. gingivalis*, *T. denticola*, and *T. forsythia* in the gingival crevices of both patients with periodontal disease and those with periodontitis associated with a depressive disorder under antidepressant treatment, before and 12 weeks after the application of the FMD protocol, in order to highlight potential differences between the two groups in terms of microbial response to therapy.

The null hypothesis was that no statistically significant differences would be found in total bacterial load or in the levels of *Aggregatibacter actinomycetemcomitans, Porphyromonas gingivalis, Treponema denticola, and Tannerella forsythia* between patients with periodontitis alone and those with periodontitis and depressive disorder, either before or after FMD.

## 2. Materials and Methods

### 2.1. Study Design, Population, and Eligibility Criteria

This study was designed as a prospective, controlled, non-randomized clinical study conducted to evaluate the effects of full-mouth disinfection (FMD) on subgingival microbiota in patients with stage II periodontitis, with and without comorbid depressive disorder. The research was conducted in accordance with the principles of the Declaration of Helsinki and was approved by the Research Ethics Committee of the “Grigore T. Popa” University of Medicine and Pharmacy, Iași, Romania (approval no. 400/15 February 2024).

Sample size calculation was performed using G*Power version 3.1 (Heinrich-Heine University, Düsseldorf, Germany), based on an expected medium effect size of 0.35 (according to Cohen’s classification), with a statistical power of 0.8 and an alpha level of 0.05. The analysis indicated a minimum sample size of 76 participants.

A total of 80 patients were recruited from the University’s Dental Education Center and affiliated private dental practices. Participants were allocated into two parallel groups based on the presence or absence of a clinically diagnosed depressive disorder, rather than through randomization: PS Group (*n* = 40) diagnosed with periodontitis without depression and PD Group (*n* = 40) diagnosed with periodontitis associated with depressive disorder under antidepressant treatment. All participants provided written informed consent prior to enrollment.

All included patients were diagnosed with moderate periodontitis, based on clinical and radiographic findings, in accordance with the 2017 World Workshop classification of periodontal and peri-implant diseases and conditions. This corresponds to stage II periodontitis, defined by clinical attachment loss of 3–4 mm, probing depth ≤5 mm, and radiographic bone loss limited to the coronal third of the root. Only patients with stage II periodontitis were included in order to ensure homogeneity of disease severity across the study groups.

Inclusion criteria were as follows: age between 25 and 65 years, presence of Ramfjord teeth, diagnosis of moderate (stage II) periodontitis, good cooperation, and absence of systemic diseases. For the PD group, an additional inclusion criterion was a confirmed diagnosis of depressive disorder under ongoing antidepressant therapy. The diagnosis of depressive disorder was established by qualified psychiatrists according to the Diagnostic and Statistical Manual of Mental Disorders, Fifth Edition (DSM-5) criteria. All patients were undergoing ongoing antidepressant therapy at the time of enrollment. No standardized depression severity scale was applied in this study, which represents a limitation and may have introduced heterogeneity within the depression group.

Exclusion criteria applied to both groups included: any periodontal treatment or use of systemic antibiotics within the previous 6 months, smoking, pregnancy or lactation, systemic conditions such as leukemia, cancer, AIDS, or liver disease, use of anticoagulant therapy, contraindications to ultrasonic scaling, and known allergies to amoxicillin, metronidazole, or chlorhexidine.

This selection ensured that all participants presented with a comparable level of periodontal severity and that baseline microbiological conditions were not influenced by recent antibiotic exposure.

### 2.2. Clinical Periodontal Assessment

Clinical periodontal parameters were recorded at baseline and at 12 weeks following treatment.

Bleeding on probing (BOP) was assessed at six sites (mesiobuccal, mid-buccal, distobuccal, mesiolingual, mid-lingual, distolingual) per each Ramfjord tooth (1.6, 2.1, 2.4, 3.6, 4.1, 4.4), using a calibrated periodontal probe. The presence or absence of bleeding within 15 s after gentle probing was recorded for each site. BOP was expressed as the percentage of sites exhibiting bleeding out of the total number of sites examined.

Plaque Index (IP) was evaluated using a standardized plaque scoring system, assessing the presence of visible plaque accumulation on tooth surfaces. Scores were recorded at multiple sites per tooth, and results were expressed as a percentage of surfaces with plaque.

All clinical measurements were performed by the same trained examiner to ensure consistency and reduce inter-examiner variability. The examiner was calibrated prior to the study, and intra-examiner reproducibility was assessed to ensure reliability of clinical measurements.

### 2.3. Clinical Protocol and Sample Collection

Following a comprehensive oral examination, subgingival plaque samples were collected from the six Ramfjord teeth (1.6, 2.1, 2.4, 3.6, 4.1, 4.4) at the site with the deepest probing depth. Each site was isolated using sterile cotton rolls and air-dried. Sterile paper points (06 taper, Dr. Protect) were inserted into the gingival sulcus for 30 s and subsequently transferred into 1.5 mL cryovials containing RNA Save solution (Biological Industries, Beit HaEmek, Israel). Sampling was performed at baseline (prior to treatment) and at 12 weeks post-treatment.

Subgingival sampling was performed prior to any supragingival or subgingival instrumentation in order to avoid contamination and ensure accurate baseline microbiological assessment.

### 2.4. DNA Extraction and Quantification of Periodontal Pathogens

DNA was extracted using the MagaZorb^®^ DNA Mini-Prep Kit (Promega, Madison, WI, USA), following the manufacturer’s protocol. A high-purity water sample was processed in parallel as a contamination control.

The analysis included quantification of total bacterial load and four key periodontal pathogens: *Aggregatibacter actinomycetemcomitans* (Aa), *Porphyromonas gingivalis* (Pg), *Treponema denticola* (Td), and *Tannerella forsythia* (Tf).

DNA purity and concentration were assessed using a NanoPhotometer^®^ (Implen GmbH, Munich, Germany) at OD260 nm. Purity was evaluated using the OD260/OD280 ratio, with values between 1.7 and 2.0 considered acceptable.

Quantitative PCR (qPCR) was performed using TaqMan-based real-time PCR on the Mx3005P qPCR platform (Stratagene, La Jolla, CA, USA). Pathogen-specific primers and probes targeting conserved regions of the waaA (kdtA) and waaG genes were employed [19]. Each gene was present in a single copy per genome, allowing direct conversion of amplicon counts to bacterial counts.

A recombinant plasmid (Primer Design, Eastleigh, UK) containing the target sequences served as a positive control and was serially diluted from 10^1^ to 10^7^ copies to generate standard curves for quantification. High-purity water was used as the negative control.

The lower end of the standard curve was 10 copies per reaction. Therefore, measurements approaching this range were interpreted cautiously as being close to the lower analytical sensitivity of the assay. Values falling below the detection threshold were considered undetectable and were not interpreted as complete absence of the target microorganism.

Each 25 µL qPCR reaction included 2 µL of extracted DNA, 12.5 µL of GoTaq Master Mix, 0.4 µL of ROX reference dye, primers, probes, and nuclease-free water to the final volume. Primer concentrations were 100 nM for Aa and Tf, and 300 nM for Pg and Td; probe concentrations were 200 nM for Aa and Pg, and 100 nM for Td and Tf.

Thermal cycling conditions consisted of an initial denaturation at 95 °C for 10 min, followed by 40 cycles of 95 °C for 30 s and 60 °C for 1 min.

Each sample was analyzed in duplicate to ensure reproducibility, and only consistent amplification profiles were considered for final quantification. Only amplification curves with appropriate exponential profiles and cycle threshold (Ct) values within the expected range were included in the analysis.

All baseline and post-treatment samples were processed using identical protocols, including sampling, DNA extraction, qPCR platform, reagents, and amplification conditions. Quantification was performed using the same standard curve approach for all samples, and bacterial load was expressed as genomic equivalents (DNA copy numbers) per sample obtained from subgingival paper point collections.

Negative controls showed no amplification, confirming the absence of contamination, while positive controls demonstrated consistent amplification across all runs.

A positivity threshold for each bacterial species was defined based on the analytical sensitivity of the assay. Samples with values ≥10 copies/reaction (lower limit of detection) were considered positive, while values below this threshold were classified as undetectable. Therefore, prevalence was calculated as the proportion of samples with detectable bacterial counts above this threshold.

### 2.5. Full-Mouth Disinfection (FMD) Protocol and Re-Evaluation

All patients underwent full-mouth disinfection (FMD) according to a standardized protocol [1]. The procedure included oral hygiene instruction and motivation, followed by supragingival scaling and subgingival instrumentation of all periodontal pockets within 24 h using ultrasonic scalers and Gracey curettes.

Full-mouth antiseptic disinfection was performed using 0.2% chlorhexidine solution, including mouth rinsing and tongue brushing. Patients were instructed to rinse with 0.2% chlorhexidine mouthwash two to three times daily for 14 days.

No adjunctive systemic antibiotic therapy was administered, in order to evaluate the isolated effect of mechanical and local antiseptic therapy.

Following completion of the FMD protocol, patients were re-evaluated clinically and microbiologically at 12 weeks. After reevaluation, the PS group (periodontitis without depression) was designated as RS (post-treatment), and the PD group (periodontitis with depression) as RD.

Patient adherence to oral hygiene instructions was reinforced at each visit through standardized verbal guidance. Although formal compliance monitoring was not performed, all participants received consistent instructions regarding oral hygiene practices and antiseptic use throughout the study period.

### 2.6. Statistical Analysis

All statistical analyses were performed using IBM SPSS Statistics software version 29.0.0. Descriptive statistics were calculated for all microbiological and clinical variables.

The distribution of data was assessed using the Shapiro–Wilk test, and homogeneity of variances was evaluated using Levene’s test.

Due to the wide range and skewed distribution of microbiological data, bacterial counts were log10-transformed prior to inferential analysis. Normality was reassessed after transformation, and all transformed variables met the assumptions required for parametric testing.

Between-group comparisons of microbiological variables were performed using independent samples *t*-tests (for two groups) or one-way analysis of variance (ANOVA) where appropriate. Within-group comparisons (baseline vs. post-treatment) were conducted using paired samples *t*-tests.

Bonferroni correction was applied to post hoc pairwise comparisons following ANOVA in order to reduce the risk of type I error due to multiple testing. For the five microbiological outcomes analyzed (total bacterial load, *A. actinomycetemcomitans*, *P. gingivalis*, *T. denticola*, and *T. forsythia*), the adjusted significance threshold was set at *p* < 0.01 (0.05/5). For other exploratory analyses, unadjusted *p*-values were reported and interpreted cautiously.

Clinical parameters, including bleeding on probing (BOP) and plaque index (IP), expressed as percentages, were analyzed using the same parametric approach, depending on the comparison type.

Correlation analyses were performed to evaluate the relationship between clinical parameters (BOP and IP) and microbiological variables. Pearson’s correlation coefficient was used to assess linear associations between variables.

Effect sizes were calculated to quantify the magnitude of differences, using Cohen’s d for *t*-tests and eta squared (η^2^) for ANOVA.

To account for potential confounding related to the non-randomized design, multivariable linear regression analyses were performed. Depression status was included as the main independent variable, while age and sex were entered as covariates. Smoking was controlled through the exclusion criteria.

All statistical tests were two-tailed, and a *p*-value < 0.05 was considered statistically significant. To reduce the risk of type I error due to multiple comparisons, Bonferroni correction was applied where appropriate.

## 3. Results

### 3.1. Descriptive Analysis of Microbiological and Clinical Parameters

At baseline, higher median values of total bacterial load were observed in the PD group compared to the PS group (1,190,000 vs. 78,710). Similar differences were noted for *Porphyromonas gingivalis* (205,000 vs. 9865) and *Tannerella forsythia* (148,000 vs. 19,700). Median values for *Aggregatibacter actinomycetemcomitans* and *Treponema denticola* were low in both groups, frequently at or near zero, although wider interquartile ranges were observed in the PD group.

Following the application of the Full Mouth Disinfection (FMD) protocol, median bacterial values in both RS and RD groups were 0 for all analyzed species. The interquartile ranges indicated that most post-treatment values remained at or close to the detection threshold, with occasional non-zero values observed within the upper quartiles.

The median (interquartile range) of the bacterial load across each study group, are presented in Table 1.

Log10(x + 1) transformation was applied to reduce data skewness and allow inclusion of zero values (Table 2).

### 3.2. ANOVA Comparisons

One-way analysis of variance (ANOVA) revealed statistically significant differences among the study groups for the total bacterial load as well as for each of the individual pathogens (*Aggregatibacter actinomycetemcomitans*, *Porphyromonas gingivalis*, *Treponema denticola*, and *Tannerella forsythia*), with all *p*-values < 0.001. These findings supported the rejection of the null hypotheses for all tested variables.

Subsequent post hoc pairwise comparisons (Tukey test) provided further insight into the distribution of these differences across groups. For the total bacterial load, statistically significant differences were observed between RS and PS, RS and PD, RD and PS, and RD and PD (all *p* < 0.001). A comparable pattern was noted for *A. actinomycetemcomitans*, with significantly lower values in RS and RD compared to PS (*p* = 0.001) and PD (*p* = 0.029). In the case of *P. gingivalis*, RS and RD differed significantly from both PS and PD groups (p < 0.001). For *T. denticola*, all intergroup comparisons showed statistically significant differences (*p* < 0.001), with an additional moderate but significant difference between PS and PD (*p* = 0.043). Regarding *T. forsythia*, significant differences were detected between RS and PS/PD, as well as between RD and PS/PD (*p* < 0.001), while no significant differences were found between RS and RD or between PS and PD.

The mean values ± SD clinical parameters across study groups are presented in Table 3.

At baseline, the PS group presented a mean bleeding on probing (BOP) value of 68.1% (±24.5), while the PD group showed a comparable mean of 60.6% (±20.9). Similarly, plaque index (PI) values were high in both groups, with mean values of 3.7 (±0.9) in PS and 3.9 (±0.7) in PD.

Following treatment, both clinical parameters showed marked reductions. In the RS group, the mean BOP decreased to 6.9% (±8.3), with a corresponding PI value of 1.1 (±0.9). In the RD group, even lower values were observed, with a mean BOP of 3.6% (±6.4) and a PI of 0.7 (±0.7). Overall, these values indicate a substantial decrease in both gingival inflammation and plaque accumulation after therapy in both study groups.

Within-group comparisons demonstrated statistically significant reductions in both clinical parameters following treatment. In the PS group, bleeding on probing (BOP) and plaque index (PI) decreased significantly after therapy (both *p* < 0.001). Similarly, in the PD group, both BOP and PI values were significantly reduced at the post-treatment evaluation (both *p* < 0.001).

### 3.3. Multivariable Regression Analysis

Multivariable linear regression analyses were performed to evaluate the association between depression status and microbiological parameters, adjusting for age and sex. Depression status was significantly associated with higher baseline total bacterial load (β = 0.68, 95% CI: 0.32–1.04, *p* < 0.001), as well as increased levels of Porphyromonas gingivalis (β = 0.74, 95% CI: 0.39–1.09, *p* < 0.001), *Tannerella forsythia* (β = 0.59, *p* = 0.002), and *Treponema denticola* (β = 0.41, *p* = 0.024). No significant association was observed for *Aggregatibacter actinomycetemcomitans* (*p* = 0.23). Age and sex were not significantly associated with the evaluated microbiological outcomes. The results of the regression analyses are summarized in Table 4.

### 3.4. Correlation Analysis

Pearson correlation analysis demonstrated moderate positive associations between total bacterial load and both bleeding on probing (BOP) (r = 0.52, *p* < 0.001) and plaque index (PI) (r = 0.47, *p* < 0.001). Among individual pathogens, *Porphyromonas gingivalis* and *Tannerella forsythia* showed similar moderate correlations with both clinical parameters (r ≈ 0.45–0.51, *p* < 0.001). *Treponema denticola* exhibited weaker but statistically significant correlations (r ≈ 0.32–0.36), while *Aggregatibacter actinomycetemcomitans* showed no significant association with either BOP or PI (Table 5).

**Figure 1:**
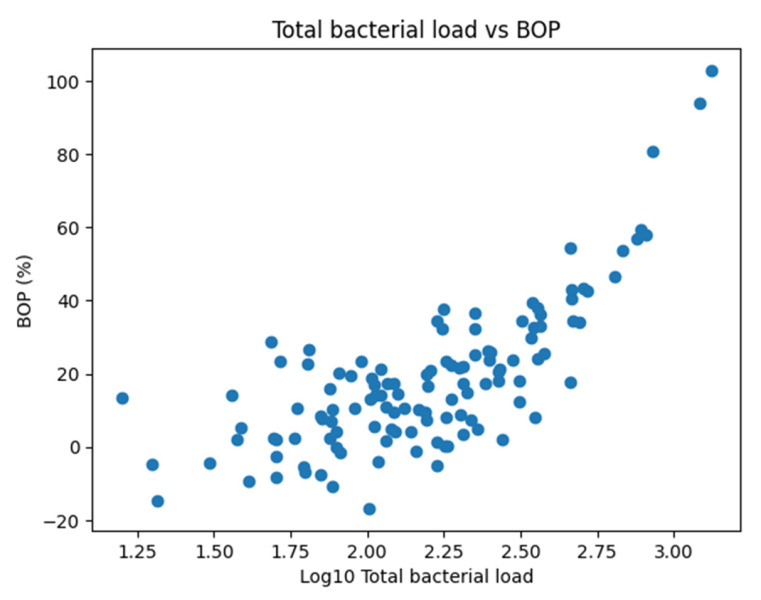
Scatter plot illustrating the relationship between log10-transformed total bacterial load (x-axis) and bleeding on probing (BOP, %) (y-axis). Each point represents an individual patient. The regression line indicates the direction and strength of the association.

**Figure 2:**
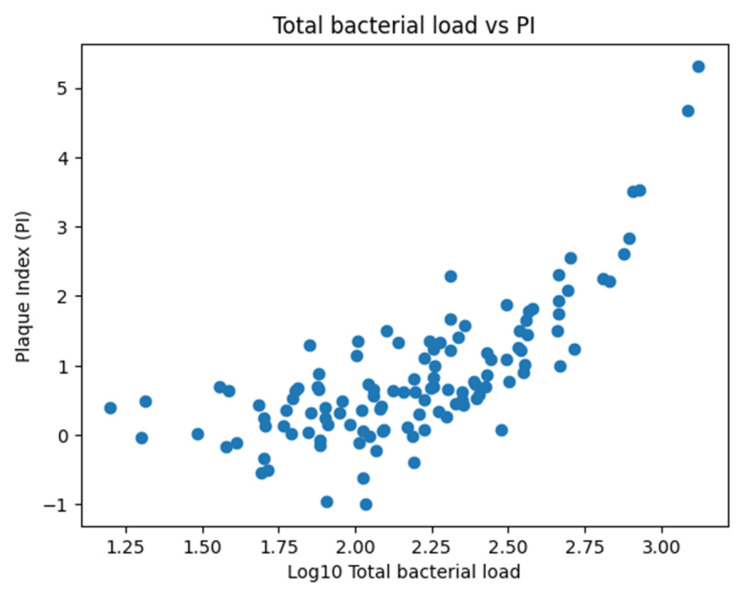
Scatter plot illustrating the relationship between log10-transformed total bacterial load (x-axis) and plaque index (PI) (y-axis). Each point represents an individual patient. The regression line indicates the direction and strength of the association.

To further explore the relationship between microbiological and clinical parameters, scatter plot analyses were performed to assess the association between total bacterial load and key clinical indicators. The distribution of individual patient values is illustrated in Figure 1 and Figure 2, allowing visualization of potential correlations between bacterial burden and both bleeding on probing (BOP) and plaque index (PI).

## 4. Discussion

The findings of the present study support the view that full-mouth disinfection (FMD), applied as a standardized non-surgical therapy, can achieve simultaneous improvement in both clinical and microbiological parameters in patients with stage II periodontitis, regardless of the presence of depressive comorbidity. Without reiterating the numerical data, a consistent pattern can be observed: following the intervention, clinical markers of inflammation and plaque control are markedly reduced, while the total bacterial load and the levels of the targeted species decline to low values, often approaching the analytical threshold of the method. This clinical–microbiological convergence is consistent with the current concept of periodontitis as a dysbiosis-driven inflammatory disease, in which reduction of the subgingival biofilm leads, through a decrease in microbial challenge, to attenuation of the local inflammatory response [20].

Although the present study focused on a targeted panel of well-established periodontal pathogens, it is important to acknowledge that periodontitis is a polymicrobial disease involving complex microbial communities. Therefore, the selected species represent key indicators of dysbiosis rather than the entire oral microbiome.

Historically, the rationale of FMD has been linked to the concept of limiting intraoral reinoculation between quadrants by treating the entire oral cavity within a short time interval, in association with antiseptic measures and rigorous hygiene control [21]. Early clinical–microbiological observations and pilot studies described a rapid reduction in subgingival pathogens and clinical improvement during the early post-therapeutic period, suggesting a potential advantage over sequential quadrant instrumentation in certain settings. However, guideline-based syntheses and systematic reviews have generally indicated that the differences between “full-mouth” strategies and the conventional approach are relatively small and depend on the protocol, adjunctive measures, and follow-up period, while the overall benefit may be comparable rather than universally superior [22]. Within this framework, the present results are consistent with the literature, showing that FMD functions as an effective intervention for controlling biofilm and inflammation without necessarily requiring systemic antibiotics to produce a favorable short-term response [23].

A clinically relevant finding was that patients with depression, who were receiving ongoing antidepressant therapy, presented a higher baseline microbial burden, particularly for taxa associated with periodontitis, without this necessarily being reflected in an obvious difference in the global clinical indicators of inflammation and plaque at baseline [24]. This apparent dissociation is plausible, since BOP and PI reflect cumulative phenomena such as plaque control, oral hygiene status, microvascular fragility, and host reactivity, whereas dysbiosis may vary in magnitude and composition between individuals without immediately producing proportional differences in overall clinical scores [22]. In addition, depression has been associated at the population level with behavioral and access-to-care factors, including less consistent oral hygiene and delayed presentation for treatment, as well as alterations in the stress–inflammation axis that may modulate susceptibility to periodontal disease. Nevertheless, the literature remains heterogeneous: some meta-analyses have shown weak or inconsistent associations between depression and periodontitis, whereas others have reported positive relationships with adverse oral outcomes, suggesting a role for residual confounding and methodological variability [25,26,27]. Therefore, interpretation should remain balanced: the data support the presence of a more heavily burdened microbial profile in a subgroup of patients with depression, but they do not demonstrate causality or a single underlying mechanism.

From the perspective of clinical parameters, the pronounced reduction in BOP following FMD suggests a robust decrease in active gingival inflammation. BOP is regarded as a sensitive indicator of local inflammation and is widely used for monitoring response to periodontal therapy and for risk stratification during maintenance. In parallel, the reduction in PI after the intervention reflects improved supragingival biofilm control, achieved through both scaling/instrumentation and the educational and motivational components of treatment. Contemporary guidelines consider these elements fundamental to the success of non-surgical periodontal therapy and to long-term stability [28]. A practical implication is that, even in vulnerable populations, including patients with affective disorders, a concentrated intervention combined with a clear hygiene plan and structured follow-up may result in clinical improvement comparable to that observed in patients without psychiatric comorbidity, provided that implementation is rigorous and behavioral support is adequate [29].

From a microbiological perspective, the reduction pattern observed for the periodontal pathogens supports the central role of periodontitis-associated communities in the initiation and maintenance of inflammation. The classical organization of taxa into subgingival “complexes” showed that the red complex, including *P. gingivalis*, *T. denticola*, and *T. forsythia*, is closely associated with clinical severity and bleeding on probing, which provides biological plausibility for the fact that these taxa are the most clearly linked to clinical parameters [30,31,32]. More recent models of dysbiosis and polymicrobial synergy further explain why reducing an entire community, rather than a single species, is relevant: periodontitis is sustained by a dysbiotic microbiota that perpetuates itself through inflammation, and effective interventions are those that reduce the burden and destabilize the pathogenic ecological network [5]. In the same context, the keystone pathogen hypothesis attributes to *P. gingivalis* the ability to disproportionately remodel the community, influence the immune response, and promote dysbiosis even when it is not numerically dominant within the biofilm [33]. These concepts help explain how reductions in *P. gingivalis* may exert downstream effects on total bacterial load and inflammatory signatures, without invoking mechanisms beyond established disease models.

The moderate positive statistical association between total bacterial load and both BOP and PI reinforces the idea that the severity of clinical inflammation and the level of plaque accumulation are, to some extent, quantifiable expressions of microbial pressure. This observation is in line with classical data showing that the structure of subgingival communities correlates with clinical indicators such as pocket depth and bleeding, as well as with more recent literature emphasizing that periodontitis is a microbiota-driven but host-mediated disease [34]. At the same time, the fact that these relationships are moderate rather than perfect is clinically important: inflammation and plaque accumulation are influenced by behavioral variables, hygiene quality, local anatomy, salivary flow, and immune phenotype, while bacterial load measured by qPCR reflects bacterial DNA and not necessarily viable bacteria or metabolic activity. Consequently, the observed correlations should be interpreted as indicators of biological coherence and clinical relevance rather than as exhaustive predictive tools [35].

The species-level correlation pattern also provides useful clarification regarding disease biology in stage II periodontitis. The stronger correlations observed for *P. gingivalis* and *T. forsythia* are consistent with their established roles in dysbiosis and inflammation: *P. gingivalis* has frequently been described as a keystone or community-activating species, whereas *T. forsythia* has been associated with periodontal lesions and proteolytic mechanisms that may amplify tissue damage [36,37]. *T. denticola* tended to show more modest associations, which is plausible given that spirochetes may act more strongly within consortia and that their clinical contribution may depend on the co-presence of other taxa and niche conditions; moreover, the virulence literature describes a wide range of factors supporting invasion and immune evasion, without these necessarily being reflected linearly in clinical scores [38]. By contrast, the absence of a significant association for *A. actinomycetemcomitans* is compatible with the epidemiological profile of this species, which is more closely linked to aggressive or localized forms and specific phenotypes, including leukotoxin-producing lineages, whereas in moderate adult periodontitis its frequency and abundance may be lower, limiting its clinical relevance in the present cohort [39]. This finding may therefore be viewed as indirect support for the appropriateness of the selected bacterial panel, as the pathogens with greater clinical relevance for moderate disease were also those that best tracked clinical variability.

The observed increase in obligate anaerobic Gram-negative periodontal pathogens in relation to the severity of clinical parameters is consistent with the well-established ecological shift within the periodontal pocket. As inflammation progresses and pocket depth increases, oxygen tension decreases, creating a favorable environment for anaerobic species. These microorganisms, including key periodontopathogens, contribute to a self-sustaining cycle of dysbiosis and inflammation through virulence factors that promote tissue breakdown and immune dysregulation. Therefore, the association between higher bacterial load and more pronounced clinical inflammation reflects not only a quantitative increase in microbial burden but also a qualitative shift toward a more pathogenic biofilm.

From the perspective of clinical practice, the findings suggest two complementary directions of application. The first concerns FMD as a useful logistical and therapeutic strategy in populations for whom adherence to multiple visits may be difficult, including individuals with depression, by compressing active treatment into a short time frame and reducing the window for intraoral reinoculation; this approach is supported by both the conceptual literature and evidence syntheses showing outcomes comparable to conventional instrumentation when the protocol is applied rigorously [40]. The second concerns the integration of periodontology with behavioral medicine: because depression is frequently associated with unfavorable oral outcomes and reduced use of dental services, interventions could incorporate basic screening for risk behaviors, adapted communication, simplified oral hygiene plans, and more structured maintenance schedules [41]. For future research, it would be useful to stratify patients according to depression severity and antidepressant treatment type, to include local and systemic inflammatory biomarkers, and to extend microbial profiling beyond a targeted pathogen panel through metagenomic, 16S, or metatranscriptomic approaches. Longer follow-up studies would also be valuable in determining whether the initial microbiological differences associated with depression translate into different recurrence risks during the maintenance phase.

As a conclusion, the clinical outcome of FMD was satisfactory, as both inflammatory parameters and microbial burden decreased markedly after treatment, despite the absence of adjunctive systemic antibiotics. However, further improvements could potentially be achieved by integrating adjunctive approaches such as locally delivered antimicrobials, photodynamic therapy, probiotics, host-modulation strategies, or, in selected cases, systemic antibiotics, particularly in patients with high baseline microbial burden or incomplete response to conventional therapy.

Beyond statistical significance, the marked reductions in bleeding on probing, plaque accumulation, and bacterial load suggest a clinically meaningful improvement in periodontal inflammation and microbial control. However, these findings should be interpreted as evidence of short-term clinical benefit, and longer follow-up is needed to confirm the stability and long-term relevance of these improvements.

Interpretation of the present findings should be made in light of several methodological limitations that may influence generalizability. First, because group allocation was not randomized, as is often the case in studies involving psychiatric comorbidity, residual confounding cannot be excluded, including subtle differences in behavior, socioeconomic status, and adherence to hygiene or maintenance. Depression severity was not assessed using standardized scales, which limits the ability to explore dose–response relationships and may obscure potential subgroup differences. This should be considered when interpreting the association between depression and periodontal or microbiological outcomes.

Furthermore, the microbiological panel was targeted, comprising total bacterial load and a limited number of species, while qPCR detects DNA rather than viability; thus, values approaching the detection threshold should not be interpreted as biological eradication. In addition, sampling from index teeth such as Ramfjord teeth and from the deepest sites may underestimate intraoral heterogeneity, whereas a follow-up period of several weeks is more informative for early response than for long-term control. Another methodological limitation is that formal intra-examiner reproducibility values, such as ICC, were not calculated, although all clinical measurements were performed by a single trained examiner to reduce measurement variability.

From a preventive perspective, patients with depressive disorders may benefit from tailored periodontal care strategies that address both behavioral and biological risk factors. These may include more frequent recall visits, simplified and reinforced oral hygiene instructions, and motivational support to improve adherence. Management of medication-induced xerostomia, through salivary substitutes or stimulation, may help maintain oral ecological balance. In addition, interdisciplinary collaboration with mental health professionals could contribute to improved overall compliance and long-term periodontal stability in this vulnerable patient group.

## 5. Conclusions

- Full-mouth disinfection (FMD) proved to be an effective therapeutic approach in reducing the overall microbial burden in the oral environment among patients with periodontitis, regardless of the presence of comorbid depressive symptoms.
- The FMD protocol was associated with a notable decrease in the levels of major periodontal pathogens, including *Aggregatibacter actinomycetemcomitans*, *Porphyromonas gingivalis*, *Treponema denticola*, and *Tannerella forsythia*, supporting its clinical utility in managing microbial dysbiosis.
- FMD was associated with improvements in bleeding on probing, plaque index, and subgingival microbial profiles.
- Periodontitis-associated species, particularly *P. gingivalis* and *T. forsythia*, were more closely related to clinical disease expression.
- The presence of depressive comorbidity did not appear to influence the short-term periodontal treatment response.

Overall, the observed reductions in clinical and microbiological parameters indicate not only statistically significant changes but also clinically relevant short-term improvements in periodontal inflammation and microbial control following FMD.

## Figures and Tables

**Table 1:** Median (interquartile range) bacterial load across study groups.

Group	Total	AA	PG	TD	TF
PS	78,710 (23,265–481,425)	0 * (0–3895)	9865 (1070–136,000)	0 * (0–4820)	19,700 (2050–79,400)
PD	1,190,000 (236,250–4,876,900)	0 * (0–14,700)	205,000 (48,800–1,950,000)	2790 (0–79,200)	148,000 (17,500–1,070,000)
RS	0 (0–4821) *	0 (0–167) *	0 (0–3204) *	0 (0–211) *	0 (0–2543) *
RD	0 (0–6711) *	0 (0–281) *	0 (0–4966) *	0 (0–1470) *	0 (0–11,549) *

Abbreviations: PS—periodontitis without depression (pre-treatment); PD—periodontitis with depression (pre-treatment); RS—periodontitis without depression (post-treatment); RD—periodontitis with depression (post-treatment); AA—*Aggregatibacter actinomycetemcomitans*; PG—*Porphyromonas gingivalis*; TD—*Treponema denticola*; TF—*Tannerella forsythia*. Note: Data are presented as median (interquartile range, Q1–Q3), calculated from individual patient values within each group. * Values represent measurements below the detection limit.

**Table 2:** Log10-transformed mean bacterial load across study groups.

Group	Total	AA	PG	TD	TF
PS	5.70	1.20	4.95	2.10	4.60
PD	6.55	1.35	5.80	3.20	5.40
RS	0.20	0.00	0.15	0.05	0.18
RD	0.25	0.00	0.20	0.10	0.30

Note: Values represent mean log10(x + 1)-transformed bacterial counts.

**Table 3:** Mean ± SD clinical parameters across study groups.

Group	BOP (%)	PI
PS	68.1 ± 24.5	3.7 ± 0.9
PD	60.6 ± 20.9	3.9 ± 0.7
RS	6.9 ± 8.3	1.1 ± 0.9
RD	3.6 ± 6.4	0.7 ± 0.7

**Table 4:** Multivariable linear regression analysis of baseline microbiological parameters.

Outcome Variable	Predictor	β (Coefficient)	95% CI	*p*-Value
log Total load	Depression	0.68	0.32–1.04	<0.001
	Age	0.01	−0.01–0.03	0.21
	Sex	0.12	−0.25–0.49	0.52
log Pg	Depression	0.74	0.39–1.09	<0.001
	Age	0.00	−0.02–0.02	0.88
	Sex	0.15	−0.20–0.50	0.40
log Tf	Depression	0.59	0.21–0.97	0.002
	Age	0.01	−0.01–0.03	0.27
	Sex	0.10	−0.28–0.48	0.60
log Td	Depression	0.41	0.05–0.77	0.024
	Age	0.00	−0.02–0.02	0.95
	Sex	0.08	−0.30–0.46	0.68
log Aa	Depression	0.18	−0.12–0.48	0.23
	Age	0.00	−0.01–0.01	0.89
	Sex	0.05	−0.20–0.30	0.71

Abbreviations: Pg—*Porphyromonas gingivalis*; Tf—*Tannerella forsythia*; Td—*Treponema denticola*; Aa—*Aggregatibacter actinomycetemcomitans*.

**Table 5:** Pearson correlation between bacterial load and clinical parameters (BOP and PI).

Variable	BOP (r)	*p*-Value	PI (r)	*p*-Value
Total	0.52	*p* < 0.001	0.47	*p* < 0.001
AA	0.18	*p* = 0.12	0.15	*p* = 0.18
PG	0.49	*p* < 0.001	0.45	*p* < 0.001
TD	0.36	*p* = 0.004	0.32	*p* = 0.009
TF	0.51	*p* < 0.001	0.48	*p* < 0.001

## Data Availability

All the data presented in this study are available within the article.

## Abbreviations

The following abbreviations are used in this manuscript:

FMD	Full-mouth disinfection
FMS	Full-mouth scaling
SRP	Scaling and root planing
qPCR	Quantitative polymerase chain reaction
AA	*Aggregatibacter actinomycetemcomitans*
PG	*Porphyromonas gingivalis*
TD	*Treponema denticola*
TF	*Tannerella forsythia*
SSRI	Selective serotonin reuptake inhibitor
SNRI	Serotonin–norepinephrine reuptake inhibitor
TCA	Tricyclic antidepressant
MAOI	Monoamine oxidase inhibitor
DNA	Deoxyribonucleic acid
OD	Optical density
Ct	Cycle threshold
